# Multinational, observational study of procalcitonin in ICU patients with pneumonia requiring mechanical ventilation: a multicenter observational study

**DOI:** 10.1186/cc10087

**Published:** 2011-03-07

**Authors:** Frank Bloos, John C Marshall, Richard P Dellinger, Jean-Louis Vincent, Guillermo Gutierrez, Emanuel Rivers, Robert A Balk, Pierre-Francois Laterre, Derek C Angus, Konrad Reinhart, Frank M Brunkhorst

**Affiliations:** 1 Department of Anesthesiology and Intensive Care Medicine, Jena University Hospital, Erlanger Allee 101, 07747 Jena, Germany; 2 Department of Surgery, Li Ka Shing Knowledge Institute, St Michael's Hospital, University of Toronto, 30 Bond Street, Toronto, ON M5B 1W8, Canada; 3 Division of Critical Care Medicine, Department of Medicine, Cooper University Hospital, One Cooper Plaza D393, Camden, NJ 08103, USA; 4 Department of Intensive Care, Erasme University Hospital, Route de Lennik 808, 1070 Brussels, Belgium; 5 Division of Pulmonary and Critical Care Medicine, The George Washington University, 2150 Pennsylvania Ave., Washington, DC 20037, USA; 6 Department of Critical Care Medicine, Henry Ford Hospital, 2799 West Grand Boulevard, Detroit, MI 48202, USA; 7 Department of Pulmonary Critical Care, Medicine Rush Presbyterian St. Luke's Medical Center, 1753 West Congress Parkway, Chicago, IL 60612-3809, USA; 8 Department of Intensive Care, Cliniques Universitaires St-Luc Ave., Hippocrate 10, 1200 Brussels, Belgium; 9 Department of Critical Care Medicine, University of Pittsburgh, 3550 Terrace Street, Pittsburgh, PA 15261, USA

## Abstract

**Introduction:**

The intent of this study was to determine whether serum procalcitonin (PCT) levels are associated with prognosis, measured as organ dysfunctions and 28-day mortality, in patients with severe pneumonia.

**Methods:**

This was a multicenter, observational study of critically ill adult patients with pneumonia requiring mechanical ventilation conducted in 10 academic hospitals in Canada, the United States, and Central Europe. PCT was measured daily for 14 days using an immuno-luminometric assay.

**Results:**

We included 175 patients, 57 with community acquired pneumonia (CAP), 61 with ventilator associated pneumonia (VAP) and 57 with hospital acquired pneumonia (HAP). Initial PCT levels were higher in CAP than VAP patients (median (interquartile range: IQR); 2.4 (0.95 to 15.8) vs. 0.7 (0.3 to 2.15), ng/ml, *P *< 0.001) but not significantly different to HAP (2.2 (0.4 to 8.0) ng/ml). The 28-day ICU mortality rate for all patients was 18.3% with a median ICU length of stay of 16 days (range 1 to 142 days). PCT levels were higher in non-survivors than in survivors. Initial and maximum PCT levels correlated with maximum Sequential Organ Failure Assessment (SOFA) score r^2 ^= 0.50 (95% CI: 0.38 to 0.61) and r^2 ^= 0.57 (0.46 to 0.66), respectively. Receiver operating curve (ROC) analysis on discrimination of 28-day mortality showed areas under the curve (AUC) of 0.74, 0.70, and 0.69 for maximum PCT, initial PCT, and Acute Physiology and Chronic Health Evaluation (APACHE) II score, respectively. The optimal cut-off to predict mortality for initial PCT was 1.1 ng/ml (odds ratio: OD 7.0 (95% CI 2.6 to 25.2)) and that for maximum PCT was 7.8 ng/ml (odds ratio 5.7 (95% CI 2.5 to 13.1)).

**Conclusions:**

PCT is associated with the severity of illness in patients with severe pneumonia and appears to be a prognostic marker of morbidity and mortality comparable to the APACHE II score.

## Introduction

Respiratory tract infections requiring mechanical ventilation account for the majority of all infections treated in the intensive care unit (ICU) and are associated with prolonged hospital stay and high ICU mortality [1-3]. The Pneumonia Severity Index (PSI) is commonly used for risk stratification of patients with pneumonia. However, this parameter showed only moderate association with outcome prediction and was judged to be inadequate to guide clinical care [4].

Numerous studies have evaluated the diagnostic performance of invasive procedures, or of biochemical and molecular markers in blood or bronchoalveolar lavage (BAL) in patients with ventilator-associated pneumonia (VAP), hospital acquired pneumonia (HAP) and community acquired pneumonia (CAP). These methods are difficult to apply to daily clinical practice and none has proved to be predictive of outcome [5-8]. Furthermore, many aspects in the strategies for diagnosing HAP and VAP especially regarding the importance of invasive procedures are still controversial [9,10]. Indeed, a recent study revealed that use of invasive procedures for etiological diagnosis of pneumonia varies considerably between European ICUs [11]. This uncertainty is most likely responsible for antibiotic overtreatment observed in this group of patients [12,13]. Thus, measures to aid the early identification of patients with pneumonia are underdeveloped. Such measures are needed as patients with pneumonia are at high risk of death and would benefit from early adaption of therapy.

Procalcitonin (PCT), a relatively novel marker of infectious processes, has been shown to be associated with the severity of inflammation and prognosis during sepsis and septic shock [14-16]. In two large studies in the emergency department, low PCT-values were associated with a low risk of death in patients with CAP [17,18]. Luyt and colleagues reported that PCT levels decreased during the clinical course of VAP but were significantly higher from Day 1 to Day 7 in patients with unfavorable outcomes [19]. The significance of PCT is emphasized by the observation that the course of PCT levels may safely guide antimicrobial therapy in patients with community acquired lower respiratory tract infections [20,21] and ICU patients with suspected bacterial infections [22]. However, data about the significance of PCT in patients with hospital and ventilator acquired pneumonia requiring intensive care therapy are still limited.

The aim of this multicenter study was to test the hypothesis that serum PCT levels can assist in identifying patients with severe pneumonia who are at increased risk of poor outcome, measured as organ dysfunction and 28-day mortality.

## Materials and methods

In this multicenter, multi-national, observational study, patients admitted consecutively to the ICUs of 10 academic hospitals (8 in Canada and the United States and 2 in Europe) between 1 January 2003 and 20 November 2004 were screened for eligibility. The study protocol had been reviewed and approved by the Food and Drug Administration (protocol PCT-7; file number # I010023). Patients 18 years of age and older requiring mechanical ventilation with the new diagnosis of pneumonia within the last 48 hours were included. We excluded patients who were enrolled in a clinical study prior to baseline PCT sampling, had cardiogenic shock, had burns greater than 20% of total body surface, or were likely to die within 48 h, and postoperative patients following bone marrow transplant (within the last 6 months), coronary artery bypass grafts (within the last 7 days), and solid organ transplants (within the last 14 days). Patients were followed for 28 days after discharge from the ICU. The study was approved by local Institutional Review Boards/Ethics Committees of each participating institution and informed consent was obtained from the patients' next of kin.

Pneumonia was defined as the presence of new or progressive infiltrate(s), consolidation, cavitation, or pleural effusion on chest radiographs and the new onset of at least two of the following signs or symptoms: 1) cough; 2) production of purulent sputum or a change in the character of sputum; 3) auscultatory findings on pulmonary exam of crackles and/or evidence of pulmonary consolidation (dullness on percussion, bronchial breath sounds); and/or 4) the presence of acute or progressive dyspnea, tachypnea, or hypoxemia. In addition, at least one of the following criteria had to be fulfilled to establish the diagnosis of pneumonia: 1) fever, defined as body temperature > 38°C (100.4°F) taken orally; > 38.5°C (101.2°F) tympanically; or > 39°C (102.2°F) rectally or via pulmonary artery (PA) catheter; and/or 2) elevated total white blood count (WBC) > 10,000/mm^3^, or > 15% immature neutrophils (bands), regardless of total WBC, or leukopenia with total WBC < 4,500/mm^3^. Microscopic examination of the Gram stained respiratory secretions had to show the presence of microorganisms, with ≥25 polymorphonuclear cells and ≤10 squamous epithelial cells per field at 100× magnification (low-power, 10× objective).

CAP [23] was defined as the occurrence of pneumonia in patients who had not resided in a long-term care facility for ≥14 days before the onset of symptoms and did not fulfill criteria of HAP, HAP [24] as pneumonia diagnosed in hospitalized patients or those residing in a long-term care facility (> 48 hours), such as a skilled nursing home facility or rehabilitation unit, or present < 7 days after a patient was discharged from the hospital with initial hospitalization of ≥3 days duration, and VAP [25] as pneumonia that developed more than 48 hours after intubation in mechanically ventilated patients who had no clinical evidence suggesting the presence or likely development of pneumonia at the time of intubation.

Within 48 hours of enrolment, we sought to establish a diagnosis of pneumonia through culture and susceptibility testing of respiratory secretions obtained by deep expectoration, nasotracheal aspiration, intubation with endotracheal suctioning, bronchoscopy with BAL or protected-brush sampling, or transtracheal aspiration. The diagnosis could also be supported by culture of samples obtained by percutaneous lung or pleural fluid aspiration, and/or single diagnostic antibody titer, (IgM), or a four-fold increase in paired serum samples (IgG) for the presumed pathogen. Patients with burns greater than 20% of total body surface, expected death within 48 h, post bone marrow transplant within the last 6 months, cardiogenic shock, cardiovascular bypass within the last 7 days, solid organ transplant within the last 14 days, or patients participating in other studies were excluded.

Key data were verified by source documents (hospital chart). Monitoring was conducted according to Good Clinical Practice (GCP) and standard operating procedures for compliance with applicable government regulations and was performed by an independent clinical research organization.

We recorded demographic data including date of birth, gender, ethnic origin, weight, and height, type of pneumonia, and admission Acute Physiology and Chronic Health Evaluation (APACHE) II score at study enrolment. Organ dysfunction status was assessed daily as described elsewhere [26] and worst values of each calendar day were reported. A modified Sequential Organ Failure Assessment (SOFA) score that excluded the Glasgow Coma Scale (GCS) was utilized.

PCT samples were collected for 14 days or until patients were discharged from the ICU and/or no longer required any mechanical ventilatory support. Blood samples not expected to be analyzed within 24 h of collection were frozen at -20°C for later analysis. PCT was measured using an immunoluminometric assay (LUMItest^®^; BRAHMS GmbH, Hennigsdorf, Germany). PCT levels were not available to the investigators until completion of the study and had no impact upon patient care during the course of the study.

### Statistical methods

The primary objective was to detect a correlation between maximum PCT and SOFA-score. A total of 180 subjects were required in order to significantly demonstrate that the correlation coefficient is above 0.2 with a power of 90%. Means ± standard deviations (SDs) or medians with interquartile ranges (IQR) are reported as appropriate. The three types of pneumonia were compared using tie-corrected exact Kruskal-Wallis tests. Pair-wise comparisons of HAP and VAP to CAP were added, based on tie-corrected exact Mann-Whitney U-tests. Odds ratios and receiver operating characteristic (ROC) curve methodology were used to judge the predictive power of PCT for outcome.

## Results

### Study population

Of the 200 enrolled in this study, 25 patients were excluded from the analysis of the data. Of these, 21 patients had incomplete sampling and four patients met exclusion criteria. The characteristics on admission of the 175 patients included in our analysis study group are presented in Table 1. Mean age was 62 years; roughly one-third had CAP, one-third had HAP, and one-third had VAP. The median hospital and ICU lengths of stay prior to enrolment were six days (range 0 to 368 days) and nine days (range 0 to 42 days), respectively.

**Table 1:** Characteristics of the study population stratified according to the type of pneumonia

	CAP *N *= 57	HAP *N *= 57	VAP *N *= 61	Over-all P	*P *(CAP versus VAP)
Age	61.8 ± 18.7	64.6 ± 15.8	60.7 ± 16.2	n.s.	n.s.
Male	35 (61.4%)	38 (66.7%)	35 (57.4%)	n.s.	n.s.
APACHE II score	26.2 ± 7.4	25.2 ± 9.4	20.1 ± 8.5	< 0.001	< 0.001
SOFA score	7.6 ± 3.5	6.9 ± 3.7	6 ± 3.5	0.044	0.005
On antibiotics	57 (100.0%)	55 (96.5%)	58 (95.1%)	n.s.	n.s.
***Coexisting diseases***					
Diabetes mellitus	14 (24.6%)	11 (19.3%)	12 (19.7%)	n.s.	n.s.
Cardiovascular disease	28 (49.1%)	24 (42.1%)	16 (26.2%)	0.032	0.010
Hypertension	3 (5.3%)	3 (5.3%)	5 (8.2%)	n.s.	n.s.
Malignancies	14 (24.6%)	20 (35.1%)	11 (18.0)%	n.s.	n.s.
COPD	13 (22.8%)	5 (8.8%)	10 (16.4%)	n.s.	n.s.
Liver cirrhosis	4 (7.0%)	1 (1.8%)	1 (1.6%)	n.s.	n.s.
Chronic renal failure	2 (3.5%)	4 (7.0%)	3 (4.9%)	n.s.	n.s.
HIV/AIDS	1 (1.8%)	2 (3.5%)	1 (1.6%)	n.s.	n.s.

There was no statistical difference between CAP vs. HAP in any of these variables. Continuous data are given as mean ± standard deviation. AIDS, acquired immunodeficiency syndrome; APACHE, Acute Physiology And Chronic Health Evaluation; CAP, community acquired pneumonia; COPD, chronic obstructive pulmonary disease; HAP, hospital acquired pneumonia; HIV, human immunodeficiency virus; n.s., not significant; SOFA, sequential organ failure assessment; VAP, ventilator associated pneumonia.

Patients with CAP had higher APACHE II and SOFA scores at inclusion than patients with VAP. Such a difference was not observed between VAP and HAP patients. The incidence of cardiovascular co-morbid conditions on admission to the ICU was lower in patients with VAP than in the other groups (Table 1). Positive cultures of the microbiological samples taken within 48 h were reported in 119 patients (67.4%). Gram-positive organisms were isolated in 75 patients (42.9%) and Gram-negative organisms in 63 patients (36.0%). The detected microorganisms are shown in Table 2. In all patients, except one patient with HAP, infection was adequately controlled on Day 3 according to the attending physician.

**Table 2:** Isolates from the specimen taken for microbiological proof of infection with 48 hours after enrolment

	CAP *N *= 57	HAP *N *= 57	VAP *N *= 61	Total *N *= 175
**Negative**	21 (36.8%)	21 (36.8%)	15 (24.6%)	57 (32.6%)
**Gram positive bacteria**	22 (38.6%)	22 (38.6%)	31 (50.8%)	75 (42.9%)
MSSA	8 (14.0%)	10 (17.5%)	16 (13.9%)	34 (11.6%)
Streptococcus spp.	8 (14.0%)	4 (7.0%)	5 (4.3%)	17 (5.7%)
MRSA	4 (7.0%)	1 (1.8%)	0 (0%)	5 (1.7%)
Enterococcus spp.	0 (0%)	0 (0%)	4 (6.6%)	4 (2.3%)
Other	2 (3.5%)	7 (12.3%)	6 (9.8%)	15 (8.6%)
**Gram negative bacteria**	22 (38.6%)	19 (33.3%)	22 (36.1%)	63 (36.0%)
Pseudomonas spp.	5 (8.8%)	5 (8.8%)	6 (9.8%)	16 (5.4%)
E. coli	5 (8.8%)	3 (5.3%)	4 (6.6%)	12 (4.1%)
Haemophilus spp.	3 (5.3%)	3 (5.3%)	4 (6.6%)	10 (3.4%)
Klebsiella spp.	2 (3.5%)	2 (3.5%)	2 (3.3%)	6 (2.0%)
Proteus spp.	0 (0%)	0 (0%)	3 (4.9%)	3 (1.7%)
Other	7 (12.3%)	6 (10.5%)	3 (4.9%)	16 (9.1%)
**Yeasts**	4 (7.0%)	9 (15.8%)	8 (13.1%)	21 (12.0%)

CAP, community acquired pneumonia; HAP, hospital acquired pneumonia; E. coli, Escherichia coli; MRSA, methicillin-resistent staphylococcus aureus; MSSA, methicillin-sensitive staphylococcus aureus; spp., species; VAP, ventilator associated pneumonia.

### Time course of PCT levels

**Figure 1:**
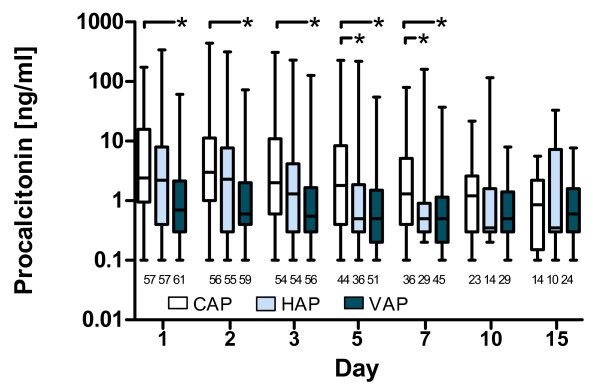
**Time course of procalcitonin levels in patients with pneumonia**. Box plot representing the time course of PCT over the two weeks following study inclusion in patients with CAP, HAP, and VAP. * *P *< 0.05 compared with CAP.

PCT levels were elevated at the time of enrolment in all groups (Table 3). Initial PCT levels were higher in CAP than VAP patients. The maximum PCT levels were higher in patients with CAP than those with HAP or VAP. Maximum PCT occurred a median of one to two days after inclusion into the study. As shown in Figure 1, PCT levels were persistently higher in patients with CAP than those with HAP during the first week following inclusion. There was no difference of initial PCT levels in culture positive and culture negative patients (1.60 (0.40 to 5.95) vs. 1.65 (0.5 to 6.9) ng/ml). Patients with positive cultures had higher maximum PCT levels (2.70 (0.65 to 8.00) vs. 2.25 (0.65 to 9.95) ng/ml). However, this difference did not reach statistical significance.

**Table 3:** Initial and maximum PCT levels, morbidity and mortality according to the type of pneumonia

	CAP *N *= 57	HAP *N *= 57	VAP *N *= 61	Overall *P*	*P* (CAP versus HAP)	*P *(CAP versus VAP)
Initial PCT (ng/ml)	2.4 (1.0, 15.8)	2.2 (0.4, 8.0)	0.7 (0.3, 2.15)	n.s	n.s.	< 0.001
Max. PCT (ng/ml)	5.3 (1.7, 17.7)	2.8 (0.4, 8.2)	1.0 (0.5, 3.4)	n.s.	0.021	< 0.001
Day of max. PCT	2 (1, 3)	1 (1, 2)	2 (1, 6)	< 0.001	0.012	n.s.
Maximum SOFA	9.5 ± 4.2	7.6 ± 3.8	6.7 ± 3.7	< 0.001	0.007	< 0.001
ICU length of stay (days)	13.0 (8.0, 17.5)	12.0 (7.0, 22.5)	26 (18, 43)	< 0.001	n.s.	< 0.001
28 days mortality n (%)	21 (36.8%)	6 (10.5%)	5 (8.2%)	< 0.001	0.002	< 0.001

Continuous data are given as median (interquartile range) or mean ± standard deviation. CAP, community acquired pneumonia; HAP, hospital acquired pneumonia; ICU, intensive care unit; PCT, procalcitonin; n.s., not significant; SOFA, sequential organ failure assessment; VAP, ventilator associated pneumonia.

### Morbidity and mortality

**Figure 2:**
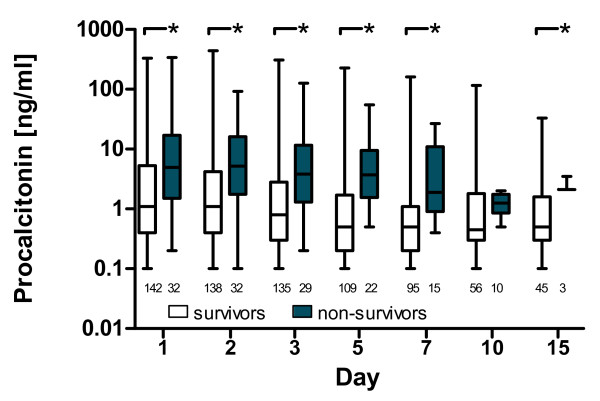
**Time course of procalcitonin levels in patients with pneumonia depending on survival**. Box plot representing the time course of PCT over the two weeks following study enrolment in survivors and non-survivors. * *P *< 0.05 compared with survivors.

**Figure 3:**
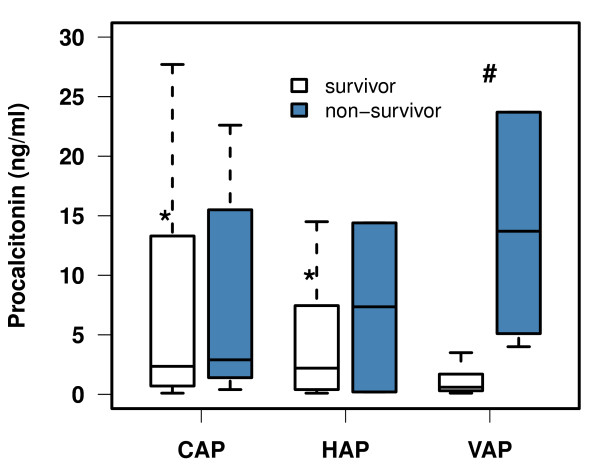
**Initial PCT-values for CAP, HAP, and VAP separated for survivors and non-survivors**. *: *P *< 0.05 (survivors vs. non-survivors), #: *P *< 0.05 (Bonferroni corrected) compared to VAP.

The overall 28-day mortality rate was 18.3% (*n *= 32) and the median ICU length of stay (LOS) was 16 (9 to 28.5) days (range 1 to 142 days). The 28-day mortality was higher in patients with severe CAP compared with those with HAP or VAP (36.8% vs. 10.5% and 8.2%, respectively, *P *< 0.01 each). Likewise, the maximum degree of organ dysfunction as assessed by the maximum SOFA score was higher in CAP compared with HAP and VAP patients. PCT levels were consistently higher in non-survivors than survivors throughout the observation period (Figure 2). Initial PCT values of VAP patients were significantly higher in non-survivors than in survivors with a median PCT of 0.6 ng/ml in the latter group (Figure 3). This difference between survivors and non-survivors was also observed in HAP but did not reach statistical significance. In the survivors, PCT values dropped to a median of 50.0% (27.3 to 100.0%) of the baseline value (*P *< 0.001) during the first five study days. A drop of similar magnitude with 53.7% (27.6 to 148.0%) was observed in the non-survivors without reaching statistical significance (*P *= 0.08).

**Figure 4:**
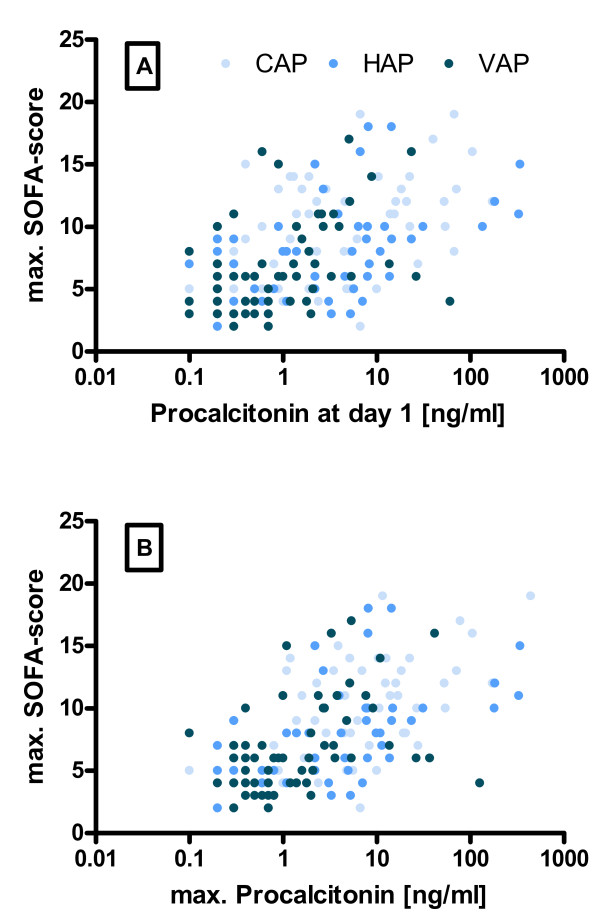
**Correlation of initial or maximum PCT with maximum SOFA-score**. Scatter plots representing the initial PCT (panel **A**) and the maximum PCT (panel **B**) vs. maximum SOFA score over the two weeks following inclusion. Square of correlation coefficients were r^2 ^= 0.50 (95% CI: 0.38 to -0.61) for initial PCT and r^2 ^= 0.57 (95% CI 0.46 to 0.66) for maximum PCT.

**Figure 5:**
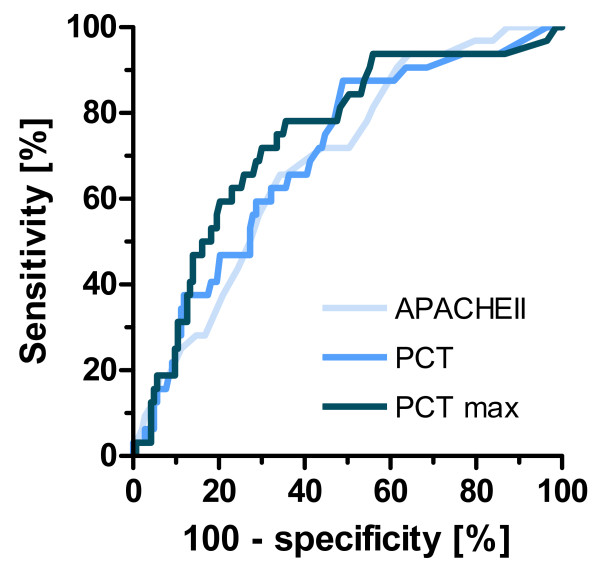
**Receiver operator characteristic (ROC) curve for 28-day mortality prediction**. Areas under the curve: maximum PCT 0.74 (95% CI: 0.65 to 0.83), initial PCT 0.70 (95% CI: 0.60 to 0.80), and APACHE II 0.69 (95% CI: 0.59 to 0.78).

Initial and maximal PCT levels correlated with maximum SOFA score (r^2 ^= 0. 51 and r^2 ^= 0.57, respectively). The association between initial and maximum PCT levels and SOFA score was independent of the type of pneumonia (Figure 4). In a ROC analysis on discrimination of 28-day mortality, the area under the curves (AUC) for maximum PCT, initial PCT, and admission-day APACHE II score were 0.74, 0.70, and 0.69, respectively (Figure 5). The AUCs were not statistically different. The best cut-off of initial PCT to predict 28-day mortality was 1.1 ng/ml (odds ratio 7.0 (95% CI 2.6 to 25.2)) and that of the maximum PCT was 7.8 ng/ml (odds ratio 5.7 (95% CI 2.5 to 13.1)). The highest AUC was observed in VAP patients with 0.71 (95% CI 0.92 to 1.01) compared to CAP with 0.41 (95% CI 0.24 to 0.92) and HAP with 0.56 (95% CI 0.58 to 0.96).

## Discussion

In this prospective multicenter study on a cohort of ICU-patients with severe pneumonia, median initial PCT levels were elevated above a normal value of 0.3 ng/ml in all groups. Those patients with ventilator associated pneumonia had the lowest initial PCT values. The maximum PCT levels were observed a median of one to two days after enrolment. Patients with severe CAP had highest initial median PCT values (2.4 ng/ml). These patients also showed greater disease severity, organ dysfunction, and mortality than HAP and VAP. This is in concordance with data from Valencia *et al.*, who reported a mortality rate of 37% in CAP patients requiring ICU therapy [27]. Median admission PCTs of 3.4 ng/ml have been observed in patients presenting with CAP in the emergency department [17].

PCT levels were higher, and remained persistently elevated, in non-survivors. Both, initial and maximum PCT values correlated with the maximum SOFA score and were a reasonable predictor of the risk of death within 28 days in these patients. In patients with severe pneumonia, initial PCT measurement allows a risk stratification similar to the APACHE II-score. The data agree with previous observations. In two studies in the emergency department with more than 1,600 patients each, PCT-values < 0.1 ng/ml in CAP were associated with a low risk of death independent of the clinical risk assessment [17,18]. PCT was also capable of identifying an unfavorable outcome in CAP patients staying at the ICU [28].

Impact of PCT-assessment is less well investigated in VAP and HAP compared to CAP. Patients with HAP not treated in an ICU have low median PCT values of 0.22 ng/ml [29]. In a single center study conducted in 44 patients with VAP, Duflo *et al*. found PCT to be significantly elevated in non-survivors: The best cut-off for serum PCT in the non-survivors in the VAP group was 2.6 ng/ml with a sensitivity of 74% and a specificity of 75% [7]. Likewise, Luyt *et al*. found high median PCT levels of about 3 ng/ml at Day 1 in patients with unfavorable outcomes during the clinical course of microbiologically proven VAP (*n *= 63) [19]. Interestingly, multivariate analyses further supported that serum PCT levels on days 1, 3, and 7 were strong predictors of unfavorable outcome [19].

We found a significant association between PCT levels and organ dysfunction as assessed by the SOFA score. Similar observations were reported by Meisner *et al. *[30] and by Schroder *et al*. in surgical critically ill patients [31]. Hedlund *et al*. showed that the severity of disease measured by the APACHE II score was strongly associated with admission levels of PCT in 96 adult patients with CAP [32]. In 110 patients with CAP, Boussekey *et al*. found higher PCT levels in bacteremic patients and/or septic shock patients (4.9 ng/ml vs. 1.5 ng/ml) and in patients who developed infection-related complications (septic shock, multiorgan dysfunction, acute respiratory distress syndrome and disseminated intravascular coagulation) during their ICU stay [33].

The association of PCT with morbidity and mortality may be of clinical importance not primarily for outcome prediction but to monitor success of therapy. Current data support the hypothesis that a drop in PCT levels represents an adequate antimicrobial therapy and may actually define a time point where antibiotic treatment can be safely withdrawn [20,21]. Recently, this has been demonstrated in ICU patients with suspected bacterial infection at admission or during their ICU stay [22]. More than 70% of these patients had pulmonary infections. Unsuccessful source control and poor outcome is associated with persistently elevated PCTs which should negatively affect outcome [14,34]. Thus, increasing PCT or persistently elevated PCT values should trigger a change in antimicrobial therapy.

In this study of severe pneumonia in mechanically ventilated patients, there was no difference in PCT levels between culture positive and culture negative pneumonia. In another study on patients with severe pneumonia as defined by a high Pneumonia Severity Index (PSI), PCT correlated with outcome but could not differentiate between bacterial and nonbacterial etiology of pneumonia [35]. In 72 children with CAP, Moulin *et al*. found PCT levels > 2 ng/ml in all 10 patients with blood culture positive for *S. pneumoniae*; PCT concentration was greater than 1 ng/ml in 86% of patients with bacterial infection, with the highest percentage being in those with positive blood culture [36]. This PCT-threshold was more sensitive and specific than CRP, IL-6, or white blood cell count for differentiating bacterial and viral causes of pneumonia. Likewise, Boussekey *et al*. found higher PCT levels in microbiologically documented CAP (median 4.9 ng/ml vs 1.5 ng/ml if no bacteria were found), but PCT levels could not discriminate between specific bacterial agents [33]. Duflo *et al*. identified VAP based on a positive quantitative culture of 10^3 ^colony-forming units/ml or more obtained *via *a mini-bronchoalveolar lavage.

Median PCT values of VAP survivors at baseline were 0.6 ng/ml in this study. This low PCT value questions the validity of currently used VAP diagnostic criteria. Luyt *et al*. found a similar low PCT of about 0.5 ng/ml in VAP survivors and doubted the usefulness of this parameter for diagnosis of VAP [19,37]. The 28-day mortality of 8.2% in patients with VAP in our study was very low. The Canadian Critical Care Trials group recorded an overall 28 days mortality rate of 18.7% in a large cohort of patients where VAP was diagnosed using similar criteria as in our study [5]. However, mortality rates between 9.8 and 93.3% have been observed depending on the presence of risk factors such as coexisting diseases, presence of bacteremia, arterial hypotension, or ARDS [38]. The low mortality rate of VAP patients and low PCT-values in the VAP survivors in this study may reflect the uncertainty in correctly diagnosing VAP despite the requirement for a positive Gram stain of respiratory secretion. Although VAP is the most frequent cause of death in hospital for patients with respiratory failure [39,40], the diagnosis of VAP is difficult. The optimal invasive procedure for diagnosing HAP or VAP remains poorly defined [9,10]. Indeed, one study demonstrated that 29% of clinically suspected VAP cases were disproved by autopsy results [41]. In this study, microbiological proof of infection was possible in about 67% of the patients. This is in good agreement with findings in large sepsis trials where microbiological proof was possible in 41 to 51% of the patients with airway infections [42,43].

It should be noted that the immunoluminometric assay for PCT measurement applied in this study has been replaced today by more modern techniques with a higher accuracy especially in the low range of PCT levels. Such accuracy is a prerequisite when using PCT for antibiotic stewardship [20]. This study was focused on high PCT concentrations for their association with mortality and organ dysfunction. It is unlikely that such a relationship is affected by the type of assay.

Measurement of PCT levels in addition to the clinical judgement may offer a solution for this diagnostic dilemma since our data suggest that baseline PCT levels greater than 1.1 ng/ml identify a group of ICU patients with a high risk to develop multiorgan dysfunction followed by death. The quality of mortality prediction was similar to the APACHE II score. These data confirm the observation by *Luyt et al.*, who found a PCT threshold of 1 ng/ml to predict unfavorable outcome [19].

Furthermore, non-survivors showed no decrease in PCT suggesting that pneumonia remained uncontrolled. Assessing adequacy of antimicrobial therapy was not part of the study hypothesis and would have been beyond the scope of this trial. However, PCT measurement offers the possibility of being a marker for monitoring therapeutic success or failure, since successful therapy is associated with a decrease in PCT levels. A PCT guided algorithm has been shown to reduce duration of antibiotic therapy without affecting patients' safety [22,44].

## Conclusions

In patients with severe pneumonia (CAP, VAP, HAP), PCT is associated with the severity of illness and is a good prognostic marker of morbidity and mortality in patients with pneumonia in demand of mechanical ventilation. The severity of illness as reflected by the degree of organ dysfunction may be a more important determinant of PCT levels than the type or cause of pneumonia.

## Key messages

• Procalcitonin (PCT) concentrations are associated with the severity of illness in patients with severe pneumonia in demand of mechanical ventilation.

• PCT is a good prognostic marker of morbidity and mortality in these patients.

• The severity of illness as reflected by the degree of organ dysfunction may be a more important determinant of PCT levels than the type or cause of pneumonia.

## Abbreviations

APACHE II: Acute Physiology and Chronic Health Evaluation II; AUC: area under the curve; BAL: bronchoalveolar lavage; CAP: community acquired pneumonia; CI: confidence interval; GCP: Good Clinical Practice; GCS: Glasgow Coma Scale; HAP: hospital acquired pneumonia; ICU: intensive care unit; IQR: interquartile range; PCT: procalcitonin; PSI: pneumonia severity index; SD: standard deviation; SOFA: Sequential Organ Failure Assessment; ROC: receiver operating characteristic; VAP: ventilator associated pneumonia; WBC: white blood cell count.

## Competing interests

FB received a speaker fee from BRAHMS. ER receives research support from the National Institute of Allergy and Infectious Disease and the Aggennix Corporation and has served as one-time consultant for Aggennix Corporation, Eisai Pharmaceuticals, Idaho Technologies and Astra Zeneca. RB has received research support, consulting fees, and honoraria from BRAHMS and from bioMerieux. DA has received consultant fees from BRAHMS, performed PCT assays for the PCT-7 trial, and had access to equipment and assays by BRAHMS as part of NIH-funded studies. KR has received consultant fees from BRAHMS. FMB has received consultant and speaker fees and grant/research support from BRAHMS. JM, RD, JV, GG and PL declare that they have no competing interests.

## Authors' contributions

FB participated in the local conduct of the trial, took part in the interpretation of the results, and drafted the manuscript. JM, RD, JV, GG, ER, RB, PL, DA and KR helped to design the study, were responsible for the conduct of the trial, and helped to draft the manuscript. FMB conceived and designed the study and helped to draft the manuscript. All authors read and approved the final manuscript.
